# Prognostic Role of Plasma PD-1, PD-L1, pan-BTN3As and BTN3A1 in Patients Affected by Metastatic Gastrointestinal Stromal Tumors: Can Immune Checkpoints Act as a Sentinel for Short-Term Survival?

**DOI:** 10.3390/cancers13092118

**Published:** 2021-04-27

**Authors:** Daniele Fanale, Lorena Incorvaia, Giuseppe Badalamenti, Ida De Luca, Laura Algeri, Annalisa Bonasera, Lidia Rita Corsini, Chiara Brando, Antonio Russo, Juan Lucio Iovanna, Viviana Bazan

**Affiliations:** 1 Department of Surgical, Oncological and Oral Sciences, Section of Medical Oncology, University of Palermo, 90127 Palermo, Italy; fandan@libero.it (D.F.); giuseppe.badalamenti@unipa.it (G.B.); ida.deluca@unipa.it (I.D.L.); laura.algeri@community.unipa.it (L.A.); annalisa.bonasera@unipa.it (A.B.); lidiarita.corsini@community.unipa.it (L.R.C.); chiara.brando@community.unipa.it (C.B.); 2 Department of Biomedicine, Neuroscience and Advanced Diagnostics (Bi.N.D.), Section of Medical Oncology, University of Palermo, 90127 Palermo, Italy; lorena.incorvaia@unipa.it (L.I.); viviana.bazan@unipa.it (V.B.); 3 Centre de Recherche en Cancérologie de Marseille (CRCM), INSERM U1068, CNRS UMR 7258, Aix-Marseille Université and Institut Paoli-Calmettes, Parc Scientifique et Technologique de Luminy, 13288 Marseille, France; juan.iovanna@inserm.fr

**Keywords:** antitumor immune response, BTN3A1, butyrophilins, circulating immune checkpoints, GIST, PD-1, PD-L1, prognostic biomarker

## Abstract

**Simple Summary:**

Recently, it was shown that circulating PD-1 and PD-L1 are correlated with shorter survival in individuals with various types of solid tumors, including lung cancer and gastrointestinal solid tumors. Nevertheless, the correlation between shorter survival and elevated levels of sPD-1 and sPD-L1 has not yet been studied in gastrointestinal stromal tumor (GIST) patients. Our study aimed to understand if soluble forms of immune checkpoints, such as sPD-1, sPD-L1, sBTN3A1, and pan-sBTN3As, may be predictors of survival for metastatic GIST (mGIST) patients, in order to obtain useful information about the clinical evolution of disease. Using receiver operating characteristic (ROC) analysis, the optimal concentration thresholds for each biomarker were identified to discriminate mGIST patients with short (≤36 months) versus long (>36 months) progression-free survival (PFS). Kaplan–Meier analysis revealed that patients with plasma concentrations under thresholds exhibited a median PFS about 20 months longer compared to subjects with levels above cut-offs. Additionally, the impact of different baseline covariates was evaluated through a multivariate analysis, showing that plasma levels of sPD-L1 and pan-sBTN3As below respective concentration thresholds and the absence of *KIT* exon 11 deletions or delins at codons 557 and/or 558 were important prognostic biomarkers for a longer PFS in mGIST patients.

**Abstract:**

Gastrointestinal stromal tumors (GISTs) represent 1% of all primary gastrointestinal tumors. Immune surveillance is often overcome by cancer cells due to the activation of immunoregulatory molecules such as programmed death protein (PD-1) and its ligand PD-L1, and butyrophilin sub-family 3A/CD277 receptors (BTN3A). Because several studies demonstrated that tumor PD-1 and PD-L1 expression may have a prominent prognostic function, this investigation aimed to discover if soluble forms of these molecules may be useful in predicting survival of metastatic GIST (mGIST) patients. Through specific ad hoc developed ELISA assays not yet available on the market, the circulating PD-1, PD-L1, BTN3A1, and pan-BTN3As levels were examined in 30 *c-KIT* exon 11-mutated mGIST patients, prior to imatinib therapy. Using specific thresholds derived by ROC analysis, we found that high baseline levels of sPD-1 (>8.1 ng/mL), sPD-L1 (>0.7 ng/mL), sBTN3A1 (>7.0 ng/mL), and pan-BTN3As (>5.0 ng/mL) were correlated with shorter progression-free survival (PFS) and poor prognosis. Contrariwise, subjects with lower plasma concentrations exhibited a median PFS about 20 months longer than to the earlier. Finally, an additional multivariate analysis revealed that circulating levels of sPD-L1 ≤ 0.7 ng/mL and pan-sBTN3As ≤ 5.0 ng/mL, and the absence of *KIT* exon 11 deletions or delins at codons 557 and/or 558 were associated with a longer PFS in mGIST patients. Our investigation, for the first time, revealed that evaluating the plasma concentration of some immune checkpoints may help prognosticate survival in mGIST patients, suggesting their potential use as prognostic biomarkers beyond the presence of *KIT* exon 11 Del or Delins at codons 557/558.

## 1. Introduction

The most frequent mesenchymal neoplasm of the gastrointestinal (GI) tract is represented by Gastrointestinal stromal tumors (GISTs), accounting for 1% of all primary gastrointestinal tumors [1,2]. Data from National Cancer Institute’s Surveillance, Epidemiology and End Results (SEER) showed that the mean age at diagnosis is 63, although GISTs may occur in individuals under the age of 40 [3]. 

Approximately 50% of GISTs arises from the stomach, 30% occurs in the ileum or jejunum, 5% is detected in the rectum and duodenum, and less than 1% in the esophageal tract, but potentially can be located in any portion of the alimentary tract, including omentum, mesentery, and occasionally, peritoneum [4,5]. The classification of stromal and mesenchymal tumors which involve the GI tract undergone a significant change over the past 30 years. The main turning point happened thanks to the identification of CD117 antigen, which proved helpful in the distinction between GISTs, typically CD117-positive, and the other group of spindle cell malignancies originating from the GI tract, including lipomas, schwannomas, leiomyomas, and hemangiomas, which are usually CD117-negative [6]. CD117 is part of c-KIT receptor, a membrane tyrosine kinase receptor (TKR) which is encoded from the *c*-*KIT* proto-oncogene [7]. Although 80–90% of GISTs harbor *c-KIT* mutations, some do not carry them. This finding may be partially explained by the occurrence of activating alterations located at the gene which encodes for the platelet-derived growth factor receptor alpha (PDGFRα) [8]. Almost 75% of gene alterations in GISTs involves exon 11, encoding for the juxtamembrane domain of TKR, whose activation results in a ligand-independent receptor dimerization. However, *c-KIT* mutations may occur in the exons 9, 13, or 17, resulting in an altered receptor signaling activation [9]. Approximately 10% of all GISTs are considered “wild type” (WT) because of the absence of detectable pathogenic alterations in both *c-KIT* and *PDGFRα* genes [10]. Mutations in the gene that encodes for succinate dehydrogenase were found in many cases of WT tumors and are associated with approximately 85% of familial GISTs [11].

Prior to the year 2000, locally advanced or metastatic GISTs were considered chemo-resistant [12]. The discovery that mutational activation of *c-KIT* or *PDGFRα* induces uncontrolled proliferation of GIST cells determined the introduction of effective treatments targeting TKRs. Imatinib, a small molecule inhibitor of TKRs originally approved for chronic myeloid leukemia, significantly changed the prognosis of locally advanced or metastatic GISTs, blocking *c-KIT* and *PDGFRα* signaling pathway via binding to the ATP-binding pocket necessary for the receptor phosphorylation and activation [13]. Imatinib treatment meaningfully prolonged median survival of metastatic GIST patients from 18 to 57 months, determining a response in over 50% of patients [14]. However, complete responses are <10% and, despite the high efficacy of imatinib, virtually all metastatic GISTs will become resistant due to additional acquired mutations in *c-KIT* [15].

Immune surveillance is often overridden by cancer cells due to the activation of immunomodulatory molecules such as programmed death protein (PD-1) and its ligand PD-L1, Cytotoxic T-Lymphocyte Antigen 4 (CTLA-4), and butyrophilin sub-family 3A/CD277 receptors (BTN3A) [16,17,18,19]. In fact, in recent years, new and effective therapeutic options involving the use of drug agents targeting CTLA-4, PD-1, and PD-L1 were developed against several solid tumors, including melanoma, renal cell carcinoma (RCC), and non-small cell lung cancer (NSCLC) [20,21,22,23,24]. Additionally, several investigations showed that anti-tumor immune responses may be positively or negatively modulated by immune checkpoints different from PD-1 and PD-L1 involved in the cross-talk between cancer and immune cells [25]. Furthermore, a considerable immunoregulatory function has been recently demonstrated also for a family of transmembrane glycoproteins, named butyrophilins (BTNs), which are part of the immunoglobulin (Ig) superfamily [26,27].

Unlike other tumors, the potential function of immune checkpoints in GISTs is still to be elucidated.

The currently known prognostic parameters for the risk assessment in GIST patients are the type of mutation, tumor localization and size, and mitotic activity. Because recent evidence revealed that PD-1 and PD-L1 levels in several tumors may be relevant prognostic factors [17,28,29,30], our investigation aimed to discover if soluble forms of immunomodulatory molecules, such as PD-1, PD-L1, BTN3A1, and pan-BTN3As, may be helpful in predicting the survival of metastatic GIST (mGIST) patients, in order to obtain significant information about the clinical evolution of disease.

## 2. Results

### 2.1. Clinico-Pathological Features of Metastatic GIST Patients

Thirty mGIST patients who fulfilled the previously determined criteria (see Section 4) were enrolled from January 2015 to March 2017 at the “Sicilian Regional Center for the Prevention, Diagnosis and Treatment of Rare and Heredo-Familial Tumors” of the Section of Medical Oncology of University Hospital Policlinico “P. Giaccone” of Palermo, by determining the clinico-pathological characteristics of their tumors.

All patients harbored a *c-KIT* exon 11 pathogenic variant (PV) and were treated by first-line imatinib 400 mg/day, based on the current therapeutic strategies. We focused on *c-KIT* exon 11-mutated patients, because they represent the most common molecular subgroup in GISTs and, at the same time, show a wide variability in Progression-Free Survival (PFS) to imatinib. Sixteen patients (53.3%) harbored *c-KIT* exon 11 deletion or delection/insertion, and 14 (46.7%) carried other PV types (duplication, insertion, or single nucleotide variant). The patients were mainly male (63%) and the median age at diagnosis was 58 years (mean 57; range 33–77 years). The most frequent site of primitive tumors was the stomach (14 patients; 46.7%), followed by the small bowel (11 patients; 36.6%) and, rarely, the GIST originated from other gastrointestinal sites (5 patients; 16.7%). The patients showed mainly larger primitive tumor (diameter > 5 cm) (18 patients; 60%) and higher mitotic index (mitoses > 5/50 HPF) (*n* = 17; 56.7%). GIST with diameter ≤ 5 cm and mitoses ≤ 5/50 HPF were less frequent (40% and 43.3%, respectively).

Regarding the metastatic site, 11 out of 30 patients (36.3.%) had only hepatic metastases, 13 patients showed peritoneal metastases (43.4%), and 6 patients (20%) had both hepatic and peritoneal involvement. The clinical features and pathological parameters are shown in Table 1.

### 2.2. Discrimination between Short-Term versus Long-Term mGIST Survivors

**Figure 1:**
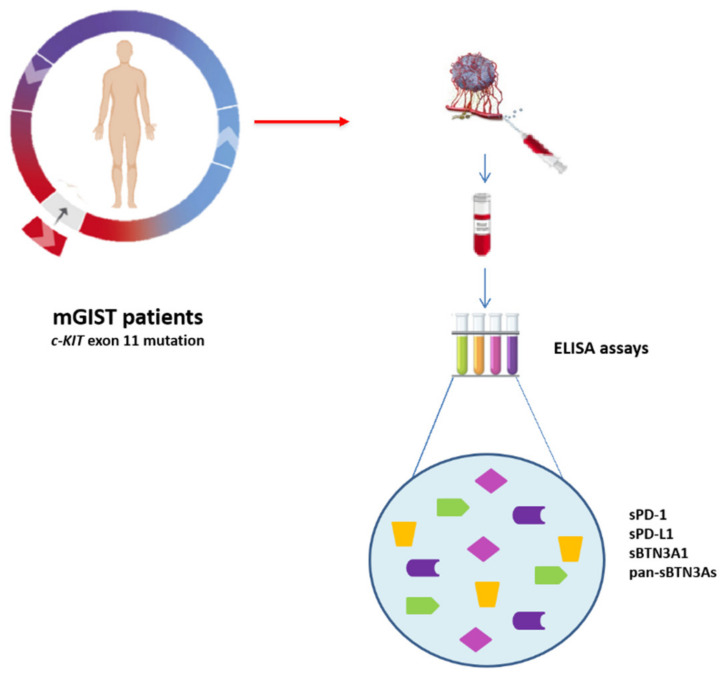
Analysis of circulating immune checkpoints in plasma from 30 *c-KIT* exon 11-mutated mGIST patients, using specific homemade ELISA tests.

The plasma PD-1, PD-L1, BTN3A1, and pan-BTN3As concentration was measured in peripheral blood from 30 mGIST patients carrying *c-KIT* exon 11 alterations, prior to imatinib therapy, using specific ad hoc developed ELISA tests not yet available on the market (Figure 1).

**Figure 2:**
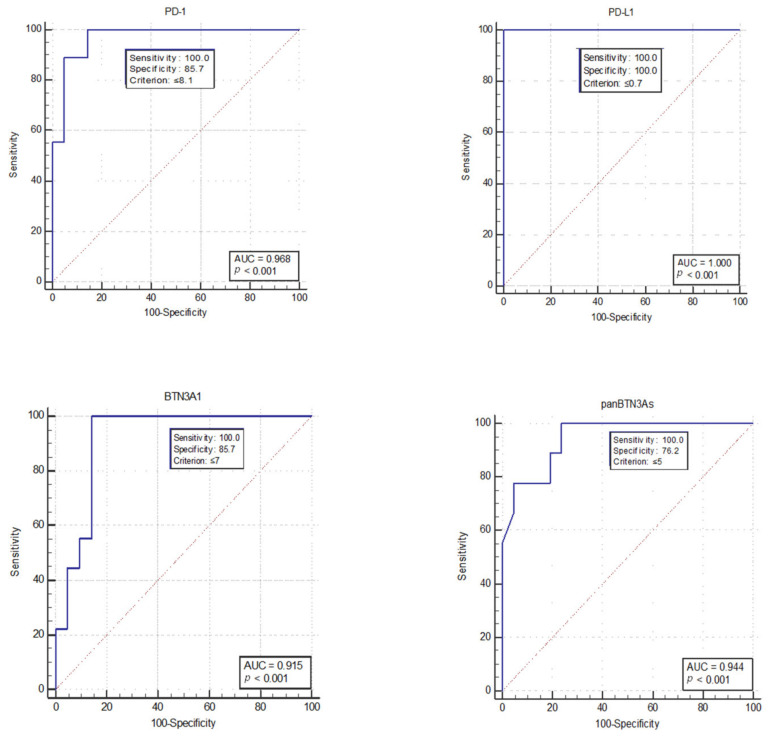
Analysis by ROC curves of circulating levels of sPD-1, sPD-L1, sBTN3A1, and pan-sBTN3As. The figure shows, for each circulating immune checkpoint, the best values of sensitivity and specificity to calculate the optimal concentration cut-offs (Youden index associated criterion). AUC, Area Under the Curve ROC. *p* < 0.001.

The receiver operating characteristic (ROC) analysis was performed to establish for each soluble biomarker the optimal concentration threshold able to discriminate short-term (≤36 months) *versus* long-term (>36 months) mGIST survivors. Short- and long-term survivors are defined as patients who showed short and long PFS, respectively, to the first-line treatment with imatinib. The ROC curve analysis showed that the best concentration threshold was 8.1 ng/mL for sPD-1 (AUC = 0.968, *p* < 0.001), 0.7 ng/mL for sPD-L1 (AUC = 1.0, *p* < 0.001), 7.0 ng/mL for sBTN3A1 (AUC = 0.915, *p* < 0.001), and 5.0 ng/mL for pan-sBTN3As (AUC = 0.944, *p* < 0.001) (Figure 2). Interestingly, these thresholds determined through ROC analysis are resulted to be close to median concentration values calculated for each soluble form of analyzed immune checkpoint, except for sBTN3A1. In fact, median concentration values were 8.91 ng/mL for sPD-1 (range 2.29 to 24.22 ng/mL), 1.06 ng/mL for sPD-L1 (range 0.30 to 2.22 ng/mL), 9.07 ng/mL for sBTN3A1 (range 0.70 to 13.53 ng/mL), and 5.66 ng/mL for pan-sBTN3As (range 0 to 9.36 ng/mL).

**Figure 3:**
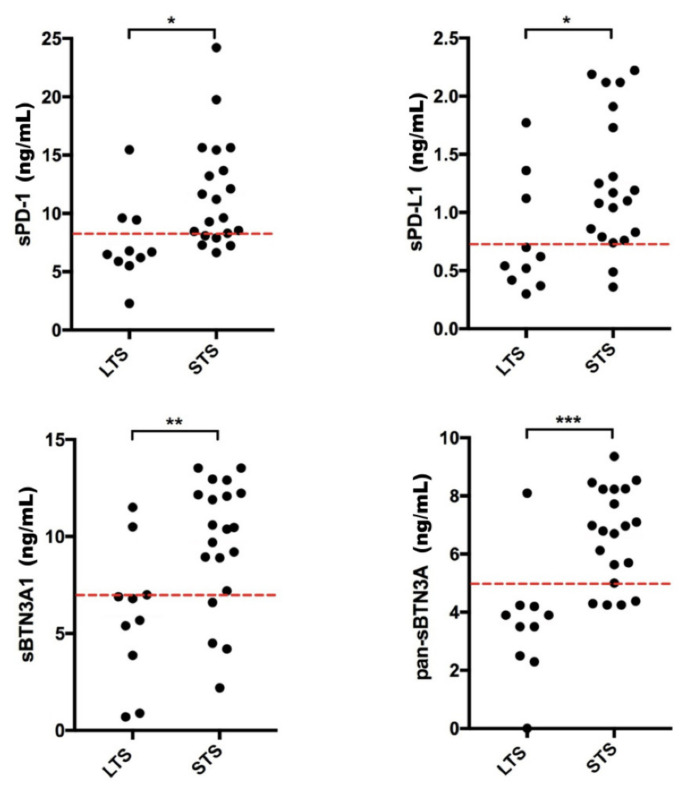
Scatter plot by group discriminating STS and LTS mGIST patients. The plasma concentrations of each soluble protein were graphically represented for short-term survival (STS) and long-term survival (LTS) patients. For each circulating immune checkpoint, the red dotted lines point out the best concentration threshold previously determined through ROC analysis. The concentrations are shown in ng/mL. * *p* < 0.05; ** *p* = 0.007; *** *p* = 0.0001.

Subsequently, the plasma concentrations of each molecule were graphically represented, discriminating and dividing into two groups the long-term survivors (LTS) from short-term survivors (STS) (Figure 3). The red dotted lines were used to point out the concentration threshold of each circulating immune checkpoint previously obtained by ROC analysis. All soluble forms showed high predictive power. Interestingly, most of mGIST patients belonging to group of LTS (PFS > 36 months) showed lower plasma levels for each soluble biomarker (sPD-1, sPD-L1, sBTN3A1, and pan-sBTN3As), whereas STS patients (PFS ≤ 36 months) predominantly exhibited higher circulating levels of these molecules.

### 2.3. High Plasma Concentrations of sPD-1, sPD-L1, sBTN3A1 and pan-sBTN3As Are Negatively Correlated with Progression-Free Survival in mGIST Patients

**Figure 4:**
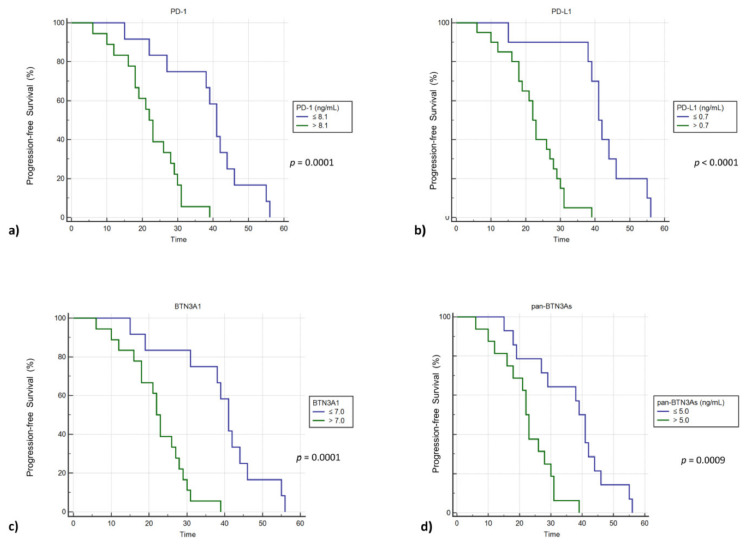
PFS analysis by Kaplan–Meier curves in 30 mGIST patients with baseline high and low plasma concentrations of sPD-1 (**a**), sPD-L1 (**b**), sBTN3A1 (**c**), and pan-sBTN3As (**d**).

Since the clinical relevance of circulating immune checkpoints as predictors of clinical outcome has yet to be defined in GIST patients, we carried out a Kaplan–Meier survival analysis in order to understand the potential prognostic value of plasma PD-1, PD-L1, BTN3A1, and pan-BTN3As in advanced GIST patients, suggesting that their plasma concentrations could be helpful, in the future, for predicting patient survival (Figure 4). Therefore, we classified the mGIST subjects with low and high plasma concentrations for each tested biomarker, using thresholds previously calculated by means of ROC analysis. Subsequently, the PFS of these patients was plotted by means of Kaplan–Meier curves (Figure 4). For each examined protein, patients with plasma concentrations above and under thresholds showed statistically significant differences in PFS. Plasma concentration cut-offs associated with unfavorable prognosis and shorter survival were determined for sPD-1 (>8.1 ng/mL), sPD-L1 (>0.7 ng/mL), sBTN3A1 (>7.0 ng/mL), and pan-sBTN3As (>5.0 ng/mL). Contrariwise, individuals with plasma concentrations under thresholds exhibited a median PFS that was about 20 months longer compared to that of subjects with levels above cut-offs. Specifically, patients with a high baseline level of sPD-1 (>8.1 ng/mL), sPD-L1 (>0.7 ng/mL), and sBTN3A1 (>7.0 ng/mL) showed a median PFS of 22 months in comparison to 41 months for subjects who exhibited lower levels of sPD-1 (log-rank *p* value = 0.0001), sPD-L1 (log-rank *p* value < 0.0001), and sBTN3A1 (log-rank *p* value = 0.0001). The same association has been observed for pan-sBTN3As (22 months vs. 39 months; log-rank *p* value = 0.0009) (Figure 4). Therefore, the soluble forms of all immune checkpoints investigated in this work have been shown to be potential survival factors in mGIST patients.

### 2.4. Multivariate Analysis of Prognostic Factors for PFS in KIT Exon 11-Mutated mGIST Patients

Since the duration of response to imatinib has been shown to be depending on the type of detected mutations in GIST patients and, in particular, individuals harboring deletions at codons 557/558 showed a significantly shorter PFS than point mutations [31], we have performed a multivariate analysis for PFS in order to correlate the type of *KIT* exon 11 mutation detected in our population cohort (Table 2), levels of plasma immune checkpoints and other clinical factors. The results of this multivariate analysis are reported in Table 3.

**Figure 5:**
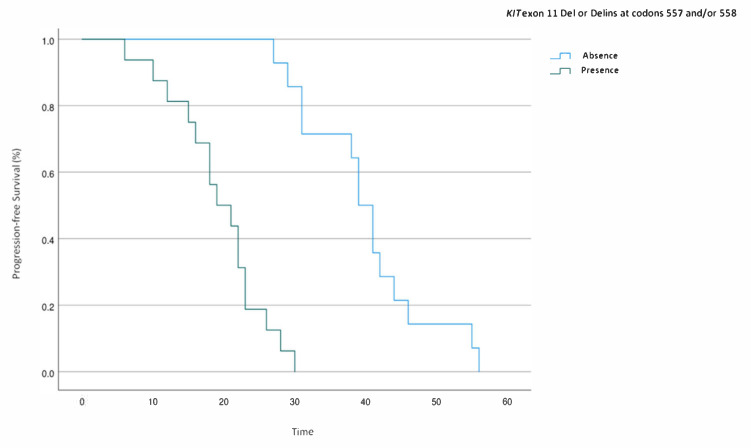
PFS analysis in mGIST patients harboring *KIT* exon 11 deletions or delins at codons 557 and/or 558.

Plasma levels of sPD-1, sPD-L1, sBTN3A1, and pan-sBTN3As, age at diagnosis, and type of *KIT* exon 11 PV were found to be statistically significantly associated with PFS in univariable analyses, while in the final multivariable Cox regression model, only the plasma levels of sPD-L1 ≤ 0.7 ng/mL (HR: 0.01; 95% CI: 0.001 to 0.18; *p* = 0.001) and pan-sBTN3As ≤ 5.0 ng/mL (HR: 4.45; 95% CI: 0.96 to 20.5; *p* = 0.05) and the absence of *KIT* exon 11 Del or Delins at codons 557 and/or 558 (HR: 0.05; 95% CI: 0.007 to 0.31; *p* = 0.003) are statistically significant. No statistically significant association was observed for other considered factors. Therefore, our analysis highlighted that the absence of *KIT* exon 11 deletions or delins at codons 557 and/or 558 (Figure 5) and expression levels of sPD-L1 ≤ 0.7 ng/mL and pan-sBTN3As ≤ 5.0 ng/mL were independent prognostic factors associated with a longer PFS in mGIST patients harboring a *KIT* exon 11 PV prior to imatinib therapy.

## 3. Discussion

In the past few years, the inhibition of the PD-1/PD-L1 immune regulatory complex led to interesting results in phase II and III clinical studies for the clinical management of various solid cancers [32,33,34,35]. Rusakiewicz et al. [36] showed that CD3+ tumor-infiltrating lymphocytes (TILs) and natural killer (NK) cells were highly activated in localized GIST patients and were located in different tumor areas, respectively, contributing independently to GIST immunosurveillance. These authors also found that immune infiltrates containing a high density of NK and CD3+ cells were associated with a longer PFS in GIST patients, regardless of the *KIT* mutational status [36]. Although it has observed that GISTs are rich in tumor-infiltrating immune cells whose presence supplies an occasion and the basis to develop efficient immunotherapy options [37,38], clinical studies assessing the effectiveness of these therapies (alone or in combination with TKIs) in GIST patients have not yielded the expected results [39,40]. Nevertheless, a preclinical study on mouse models of GIST showed that the therapeutic efficacy of imatinib can be increased by combining this TKI with immune checkpoint inhibitors targeting PD-1/PD-L1 [38].

D’Angelo et al. [41] highlighted that tumor PD-L1 expression, measured via immunohistochemistry (IHC), was greater in GISTs than in other sarcomas.

Several evidences showed that high expression of PD-1 and PD-L1 is correlated with unfavorable clinical outcome in patients with different typologies of solid cancers and lymphomas [42,43,44,45]. The same correlation was recently observed in GISTs by Zhao et al. [46], who confirmed that PD-L1 mRNA expression is a factor of unfavorable outcome associated with therapy-resistant, high-risk GIST patients. However, discordant results were reported by a previous study showing that a lower tumor PD-L1 expression was correlated with a greater risk of relapse and metastasis in localized GISTs, regardless of the c-*KIT* mutational status [30].

In general, several technical limitations concerning tissue sampling, immunohistochemistry methodological approach, and employed antibodies were detected during the evaluation of PD-L1 expression by immunohistochemistry analysis in formalin-fixed paraffin-embedded (FFPE) tissue specimens. Since PD-L1 and PD-1 are dynamic molecules as well as the immune system, their tissue expression in primary tumor may not provide an overview of metastatic disease, which evolves during progression phase [22].

Recently, plasma PD-1 and PD-L1 concentrations are resulted to be correlated with shorter survival in subjects with various types of solid cancers, including lung cancer and digestive solid tumors [17,47,48,49]. Nevertheless, the correlation between short survival and increased levels of sPD-1 and sPD-L1 has not yet been studied, to date, in individuals affected by GIST. For this purpose, our investigation focused on analyzing the baseline plasma concentrations of four immunoregulatory molecules, such as sPD-1, sPD-L1, sBTN3A1, and pan-sBTN3As, associating them with survival data from 30 mGIST patients. A Kaplan–Meier survival analysis was used with the aim of correlating the plasma concentrations of immune checkpoints analyzed in this work with PFS of patients affected by mGIST. We found that plasma PD-1 and PD-L1 concentrations negatively correlated with PFS in mGIST patients. Surprisingly, the same correlation was also observed with the butyrophilin family proteins such as pan-sBTN3As and sBTN3A1. This investigation also gave us the opportunity of categorizing the mGIST patients into two groups (STS and LTS) with different outcome and PFS according to the plasma concentrations of analyzed immune checkpoints. Since increased circulating levels of immune checkpoints have been shown to be associated with unfavorable prognosis, they could be employed hereafter as potential prognostic biomarkers. Our investigation, for the first time, revealed that evaluating the plasma concentration of some immune checkpoints could contribute to prognosticate survival in patients affected by advanced GIST and consequently implement better therapeutic strategies, thus allowing to discriminate those subjects who may take advantage from personalized therapies. Therefore, using the circulating sPD-1, sPD-L1, sBTN3A1, and pan-sBTN3As levels as sentinels for obtaining information about mGIST patient survival could be useful to improve patient clinical management as well as avoid unnecessary healthcare costs.

Finally, a multivariate analysis carried out to investigate the impact of different baseline covariates on PFS showed that plasma levels of sPD-L1 ≤ 0.7 ng/mL and pan-sBTN3As ≤ 5.0 ng/mL, and the absence of *KIT* exon 11 deletions or delins at codons 557 and/or 558 were significant prognostic factors for a longer PFS in mGIST patients.

The innovation of our study was to carry out a serial study on plasma, a biological sample that can be easily obtained, repeatedly, with little invasiveness, and that provides us with a more dynamic profile of the situation of the tumor microenvironment even during therapy, thus bypassing the limitations deriving from tissue biopsy (poor dynamism, limited quantity of sample, invasiveness). In this study, the soluble immune checkpoints were detected in plasma in place of serum, since serum concentrations have been shown to be ten times lower compared to those detected in plasma from the same blood sample. However, further investigations are required to better understanding the release mechanisms of these soluble forms from cancers and/or stromal cells.

## 4. Patients and Methods

### 4.1. Study Population

This study is a prospective analysis carried out on a group of 30 patients having a pathological diagnosis of GIST, based on the morphological features and IHC for CD117 (KIT). The study cohort included subjects with advanced disease harboring *c-KIT* exon 11 mutations candidate to first-line imatinib 400 mg/day.

Blood sample collection, mutational analysis, medical treatment, and follow-up of the study group were carried out at the “Sicilian Regional Center for the Prevention, Diagnosis and Treatment of Rare and Heredo-Familial Tumors” of the Section of Medical Oncology of University Hospital Policlinico “P. Giaccone” of Palermo, which is a reference center for Soft Tissue Sarcoma and GIST patients.

Plasma from the patients was collected from January 2015 to March 2017. The specimens were obtained at baseline, prior to imatinib treatment as first-line therapy.

The clinical and pathological information collected included gender, age, prior surgery, prior adjuvant imatinib, site of origin of primary tumors, site of active disease, baseline diameter largest lesion, baseline mitosis, risk stratification, and PFS to imatinib treatment. The response to imatinib was evaluated based on the Response Evaluation Criteria In Solid Tumors (RECIST version 1.1.) [50] in the following way: progression disease (PD), stable disease (SD), partial response (PR), complete response (CR).

Tumor samples were analyzed for *c-KIT* (exons 9, 11, 13 and 17) and *PDGFRα* (exons 12, 14 and 18) mutations by means of Sanger sequencing and, if negative for *c-KIT* and *PDGFRα* alterations, further profiled for *KIT, PDGFRA, BRAF, NF1, SDH A-D, H/K/n RAS* by Next-Generation Sequencing (NGS) panel testing.

All enrolled patients signed and provided an informed consent. This investigation (study “G-Land 2017”) was approved by the Comitato Etico Palermo 1 (authorization number: 0103–2017) of the University Hospital A.O.U.P. “P. Giaccone” of Palermo. The clinical information of each recruited subject was anonymously recorded.

### 4.2. Measurement of Plasma PD-1, PD-L1, pan-BTN3As, and BTN3A1 Levels

The peripheral blood specimens from untreated GIST patients (baseline) were processed for plasma isolation and subsequently stored as previously described [16,22].

The soluble PD-1, PD-L1, pan-BTN3As, and BTN3A1 concentrations were determined in plasma using ad hoc developed enzyme-linked immunosorbent assays (ELISAs) not yet available on the market. Since some discrepancies were detected using other tests commercially available, specific ELISAs, produced by the company DYNABIO S.A. (Parc de Luminy, Marseille, France), were used based on the previously reported specifications [16,17,22]. All four ELISAs followed the same protocol as previously described [17]. Further details on the experimental protocol regarding the homemade ELISA tests are reported in the Text S1 and Table S1. Studies evaluating the plasma and serum levels of four immune checkpoints in the same blood samples demonstrated that serum concentrations were ten times lower compared to those detected in plasma. This remark revealed that probably most of tested biomarkers was apparently lost due to the clotting process. Because of this, we used only plasma samples only in our study. In addition, all samples were diluted in the ratio of 1 to 5 before ELISA test was performed, in order to avoid interference phenomena caused by the plasma matrix. All information concerning the four ELISA tests is shown in Table 4.

### 4.3. Statistical Analysis

An analysis by ROC curves [51] was performed to establish the best concentration threshold for each soluble form of immune checkpoint, with the aim to discriminate short- *versus* long-term GIST survivors. The Kaplan–Meier method and log-rank test were used to calculate patient PFS; that is, the time range between blood sampling and disease progression. Univariate and multivariate Cox proportional hazard regression models were built in order to identify significant prognostic factors for PFS.

MedCalc software v.18.2.1 for Windows (MedCalc Software, Ostend, Belgium) and GraphPad Prism software v. 9.0.0 (GraphPad Software, San Diego, CA, USA) were used to generate data. *p* values < 0.05 were considered statistically significant.

## 5. Conclusions

Our study revealed that sPD-1, sPD-L1, sBTN3A1, and pan-sBTN3As could be advantageous biomarker candidates to prognosticate survival in mGIST patients, because individuals treated with imatinib TKI with baseline immune checkpoint expression values below the respective thresholds showed an improved clinical outcome and longer PFS than those with plasma levels above the cut-offs. Therefore, circulating immune checkpoints could represent more dynamic biomarkers compared to those present on tissue and be useful as prognostic factors in mGIST patients.

## Figures and Tables

**Table 1:** Clinico-pathological characteristics of metastatic GIST patients.

Characteristic	*N* of Patients (%)
Total patients	30
Sex	
Male	19 (63.3)
Female	11 (36.7)
Age at diagnosis (y)	
Median	58
Mean	57
Range	33–77
Age groups (y)	
≤40	2 (6.6)
41–50	7 (23.4)
51–60	9 (30)
>60	12 (40)
Type of *KIT* exon 11 PV	
Deletion/delins	16 (53.3)
Duplication/insertion/SNV	14 (46.7)
Site of origin	
Gastric	14 (46.7)
Small bowel	11 (36.6)
Other GI	5 (16.7)
Primitive tumor diameter	
≤5 cm	12 (40)
>5 cm	18 (60)
Mitosis	
≤5/50 HPF	13 (43.3)
>5/50 HPF	17 (56.7)
Site of metastasis	
Liver	11 (36.6)
Peritonuem	13 (43.4)
Liver and peritoneum	6 (20)
PFS	
≤36 months	20 (66.7)
>36 months	10 (33.3)

Abbreviations: Delins, delection/insertion; PV, pathogenic variant; SNV, single nucleotide variant.

**Table 2:** *KIT* exon 11 mutations detected in mGIST patients.

Type of Mutations	Pathogenic Variant	No. Patients (%)
Deletions	*p*.W557_K558del	6 (20%)
	*p*.K550_K558del	5 (16.8%)
	*p*.E554_K558del	1 (3.3%)
	*p*.Q556_V560del	2 (6.7%)
Del/Ins	*p*.K558_V559delinsN	1 (3.3%)
	*p*.W557_V559delinsF	1 (3.3%)
SNV	*p*.W557R	4 (13.3%)
	*p*.V560D	3 (10%)
	*p*.V559D	3 (10%)
Duplication	*p*.D572_D579dup	3 (10%)
Insertion	*p*.K558_V559insS	1 (3.3%)

**Table 3:** Univariate and multivariate analysis of prognostic factors for PFS in *KIT* exon 11-mutated mGIST patients treated with first-line imatinib.

PFS	Univariate Cox Regression		Multivariable Cox Regression	
	HR (95% CI)	*p*-Value	HR (95% CI)	*p*-Value
Gender (M vs. F)	0.90 (0.42–1.98)	NS	-	-
Age (≤55 vs. >55 years)	2.25 (1.01–4.98)	0.04	0.93 (0.33–2.61)	NS
Primitive tumor diameter (≤5 cm vs. >5 cm)	0.67 (0.31–1.47)	NS	-	-
Mitosis (≤5/50 HPF vs. >5/50 HPF)	0.74 (0.34–1.62)	NS	-	-
Gastric site of origin (No vs. yes)	0.68 (0.31–1.45)	NS	-	-
sPD-L1 (≤0.7 vs. >0.7 ng/mL)	0.05 (0.01–0.25)	<0.0001	0.01 (0.001–0.18)	0.001
sPD-1 (≤8.1 vs. >8.1 ng/mL)	0.16 (0.05–0.45)	0.0001	1.85 (0.47–7.27)	NS
sBTN3 A1 (≤0.7 vs. >0.7 ng/mL)	0.14 (0.05–0.41)	0.0001	0.84 (0.17–4.23)	NS
pan-sBTN3 s (≤5.0 vs. >5.0 ng/mL)	0.23 (0.09–0.58)	<0.001	4.45 (0.96–20.5)	0.05
*KIT* exon 11 Del or Delins at codons 557 and/or 558 (No vs. yes)	0.04 (0.008–0.35)	<0.001	0.05 (0.007–0.31)	0.003

Abbreviation: NS, Not Significant.

**Table 4:** Characteristics of ELISAs for sPD-1, sPD-L1, sBTN3 A1, and pan-sBTN3 As.

Antibodies	sPD-L1	sPD-1	pan-sBTN3As *	sBTN3A1 *
Coating Ab	α-sPD-L1 1.8 + α-sPD-L1 2.1	α-sPD-1 6.4	α-sBTN3A S148	α-sBTN3A1 S240
Detection Ab (biotinylated)	Α-sPD-L1 1.3.1	α-sPD-1 3.1	α-sBTN3A 103.2	α-sBTN3A 103.2
Detection limit (pg/mL)	20	50	100	100

* Three different isoforms (A1, A2, A3) were identified for sBTN3 A. The monoclonal antibody (Ab) specific for the sBTN3A1 isoform is indicated as α-sBTN3A1 S240. Instead, all three isoforms of sBTN3A (Pan-sBTN3A assay) were simultaneous detected using two monoclonal antibodies, α-sBTN3A S148, and α-sBTN3A 103.2.

## Data Availability

Data is contained within the article or Supplementary Material. The data presented in this study are available within the article and in Table S1.

## Supplementary Materials

The following are available online at https://www.mdpi.com/article/10.3390/cancers13092118/s1, Text S1: Experimental protocol, Table S1: Plasma concentrations of PD-1, PD-L1, BTN3A1, pan-BTN3As in mGIST patients.
