# Supplementary material for: Prognostic Role of Plasma PD-1, PD-L1, pan-BTN3As and BTN3A1 in Patients Affected by Metastatic Gastrointestinal Stromal Tumors: Can Immune Checkpoints Act as a Sentinel for Short-Term Survival?
Source: Cancers (Basel). 2021 Apr 27;13(9):2118. doi: 10.3390/cancers13092118 (PMC8125172; doi:10.3390/cancers13092118)
Supplement: Supplementary file 1 [file cancers-13-02118-s001.zip › cancers-1080078-supplementary.pdf]

cancers

MDPI

Supplementary Material: Prognostic Role of Plasma PD-1, PD-L1, Pan-BTN3As and BTN3A1 in Patients Affected by Metastatic Gastrointestinal Stromal Tumors: Can Immune Checkpoints Act as a Sentinel for Short-Term Survival?

Daniele Fanale, Lorena Incorvaia, Giuseppe Badalamenti, Ida De Luca, Laura Algeri, Annalisa Bonasera, Lidia Rita Corsini, Chiara Brando, Antonio Russo, Juan Lucio Iovanna and Viviana Bazan

Text S1: Experimental Protocol

- All steps of four ELISA tests are run at Room Temperature (RT).
- Before starting assay, all plasma samples were diluted 1/5 on a final volume of 100 µL (20 µL plasma + 80 µL of dilution buffer) for each well, in order to make negligible the interference of the plasma matrix.
- The dilution buffer used to dilute the plasma samples consists of Tris/NaCl/BSA/trehalose/thimerosal.
- The plates are coated overnight with the antibody selected for capture diluted in Tris buffer and then blocked with Tris/BSA buffer.
- For the dosage, the specific recombinant protein (PD-L1/PD1/BTN3A1/pan-BTN3As) is placed in the plate with an associated control (dilutions are made in the dilution buffer described above). A negative control (named "white") is used. Samples to be tested are incubated for 3 hours at RT.
- Wash 5 times in PBS-Tween buffer.
- A biotinylated antibody (diluted in Tris buffer /rabbit serum) is added to form the "sandwich". Incubate for 30 min at RT.
- Wash 5 times in PBS-Tween buffer.
- Avidin-peroxidase (HRP) conjugate is added. Incubate for 15 min at RT.
- Add the TMB (3,3′,5,5′-Tetramethylbenzidine) substrate. Incubate for 15 min at RT in the dark.
- The reaction is stopped with H_2SO4 and OD (optical density) read at 450 nm.

Table S1. Plasma concentrations of PD-1, PD-L1, BTN3A1, pan-BTN3As in mGIST patients.

sPD-1 (ng/mL)	s-PD-L1 (ng/mL)	sBTN3A1 (ng/mL)	pan-sBTN3As (ng/mL)
2.29	0.52	0.70	0
8.10	0.49	4.20	5.00
5.90	0.42	5.40	3.90
6.50	0.62	6.80	4.20
6.79	0.54	7.00	3.50
7.24	0.36	6.60	4.30
6.70	0.70	6.90	3.50
5.50	0.37	3.87	2.30
6.20	0.30	0.89	2.50
6.65	0.74	7.19	4.38
8.53	1.04	8.94	5.63
9.46	1.36	11.50	3.90
11.67	1.31	12.95	8.24
15.63	2.12	13.53	8.23
7.90	1.91	2.20	4.25
7.30	0.86	8.90	5.70
8.45	0.79	4.50	6.70
9.60	1.12	10.50	8.10

8.30	1.10	12.90	7.10
11.20	1.73	11.90	4.25
15.46	1.77	5.68	4.24
9.29	0.83	9.21	6.12
13.68	1.19	9.70	6.98
15.44	0.76	10.59	6.79
13.21	1.25	12.07	8.46
9.63	2.19	12.16	8.53
19.77	2.22	10.47	6.97
24.22	1.08	10.38	9.36
12.11	1.17	12.24	7.72
15.63	2.12	13.53	8.23
